# Racial/Ethnic Differences in Poststroke Rehabilitation Outcomes

**DOI:** 10.1155/2014/950746

**Published:** 2014-06-15

**Authors:** Charles Ellis, Hyacinth I. Hyacinth, Jamie Beckett, Wuwei Feng, Marc Chimowitz, Bruce Ovbiagele, Dan Lackland, Robert Adams

**Affiliations:** ^1^Department of Health Sciences and Research, Medical University of South Carolina, Charleston, SC 29425, USA; ^2^College of Health Professions, Medical University of South Carolina, 77 President Street, MSC 700, Charleston, SC 29425, USA; ^3^Department of Neuroscience, Medical University of South Carolina, Charleston, SC 29425, USA

## Abstract

*Background*. Significant racial and ethnic disparities in stroke incidence, severity, and morbidity have been consistently reported; however, less is known about potential differences in poststroke rehabilitation outcomes. *Objective*. To examine racial and ethnic differences in poststroke rehabilitation outcomes. *Methods*. We completed an in-depth search of Medline and several major journals dedicated to publishing research articles on stroke, rehabilitation, and racial-ethnic patterns of disease over a 10-year period (2003–2012). We identified studies that reported rehabilitation outcomes and the race or ethnicity of at least two groups. *Results*. 17 studies involving 429,108 stroke survivors met inclusion criteria for the review. The majority (94%) of studies examined outcomes between Blacks and Whites. Of those studies examining outcomes between Blacks and Whites, 59% showed that Blacks were generally less likely to achieve equivalent functional improvement following rehabilitation. Blacks were more likely to experience lower FIM gain or change scores (range: 1–60%) and more likely to have lower efficiency scores (range: 5–16%) than Whites. *Conclusions*. Black stroke survivors appear to generally achieve poorer functional outcomes than White stroke survivors. Future studies are warranted to evaluate the precise magnitude of these differences, whether they go beyond chance, and the underlying contributory mechanisms.

## 1. Background

Stroke is a leading cause of long-term disability and the fourth leading cause of death in the US [1]. Estimates indicate that ~795,000 Americans experience a stroke each year [1]. Among those are non-Hispanic Blacks (Blacks) who are at twice the risk of first-ever stroke compared to non-Hispanic Whites (Whites) [2]. The age-adjusted risk of ischemic stroke is 0.88 in Whites, 1.49 in Hispanics, and 1.91 in Blacks [3]. Blacks are also more likely to experience a stroke at a younger age and more likely to become disabled and experience difficulties with daily living and activities [2]. Similarly, older Blacks and Hispanics are more likely to experience higher odds of one-year all-cause poststroke rehospitalization compared to Whites after adjusting for patient and hospital characteristics [4]. Interestingly, there has been a decrease in ischemic stroke incidence among Whites in the US; however the incidence of overall ischemic stroke among Blacks has remained virtually the same [5].

Studies continue to demonstrate a differential impact of stroke between racial/ethnic groups with minorities experiencing worse poststroke outcomes. This disparity gap is of major concern because Blacks and other minorities are at a greater risk of stroke, in general, develop risk for stroke, have stroke events at younger ages, and experience greater stroke severity, mortality, or residual impairments [6–12]. For example, data collected by the Centers for Disease Control (CDC) found that Black stroke survivors are more likely to have residual poststroke activity limitations (e.g., walking, bending, carrying, etc.) when compared to Whites [13]. Consequently, many stroke survivors require poststroke rehabilitation to improve the aforementioned functional limitations.

Studies designed to examine outcomes after rehabilitation exist in the current literature. However, many of those studies reporting disparities in outcomes were not designed to primarily examine the presence of disparities in postrehabilitation outcomes. For example, two recent systematic reviews have offered an increased understanding of the complex mechanisms by which racial and ethnic disparities occur among individuals with stroke. However, neither offered substantial information related to racial or ethnic differences in outcomes among stroke survivors after receiving rehabilitation. Stansbury and colleagues (2005) completed a systematic review of the ethnic disparities in epidemiology, acute care processes, and stroke outcomes [14]. The report concluded that, in addition to greater incidence, more severe stroke, and greater mortality, racial/ethnic minorities are less likely to receive tissue plasminogen activator (tPA) and diagnostic procedures critical to early stroke diagnosis and management. In that review only one study reported data related to disparities in functional outcomes after rehabilitation. A multivariate analysis of 145 stroke patients designed to measure race differences in recovery showed that Black patients had greater physical impairment at admission which continued for 90 days [6].

The second review commissioned by the American Heart Association and American Stroke Association was designed to describe the impact of race and ethnicity on epidemiology, access to care, beliefs and attitudes, and response to treatment [15]. The report concluded that excess burden exists among minorities in the presence of stroke risk factors, stroke morbidity (prevalence, incidence, and recurrence), and stroke mortality. In addition, differences existed in disease awareness, attitudes and beliefs about stroke, compliance with care, access to stroke prevention services, and access to quality stroke care. However, the report did not address or provide any information related to poststroke rehabilitation outcomes.

The purpose of this review was to examine the current literature to determine the presence or absence of racial or ethnic disparities in outcomes after stroke rehabilitation. For this review we considered any outcome measures used by rehabilitation professionals during the rehabilitation process to measure improvement after the completion of poststroke rehabilitation programs.

## 2. Method

To complete this review, the authors searched Medline using the following Medical Subject Headings ([MeSH]) terms: stroke, rehabilitation, recovery, and outcomes, and cross-searched those with race, ethnicity, and racial/ethnic groups. We completed a separate in-depth search of the following journals using the same MeSH terms: Archives of Physical Medicine and Rehabilitation, American Journal of Physical Medicine and Rehabilitation, Ethnicity & Disease, PM&R, Neural Repair and Neurorehabilitation, and Topics in Stroke Rehabilitation and Stroke.

We limited our search to papers published over a ten-year period (2003 to 2012) and only considered papers including a US patient population and written in English. We considered randomized controlled trials, quasirandomized controlled trials, and retrospective data analyses and published scientific conference presentations that reported rehabilitation outcomes between at least two racial or ethnic groups. Because of the heterogeneity of studies, patient populations, and rehabilitation settings, we decided a priori not to perform a meta-analysis but instead to perform a qualitative analysis of the study findings.

## 3. Results

**Figure 1:**
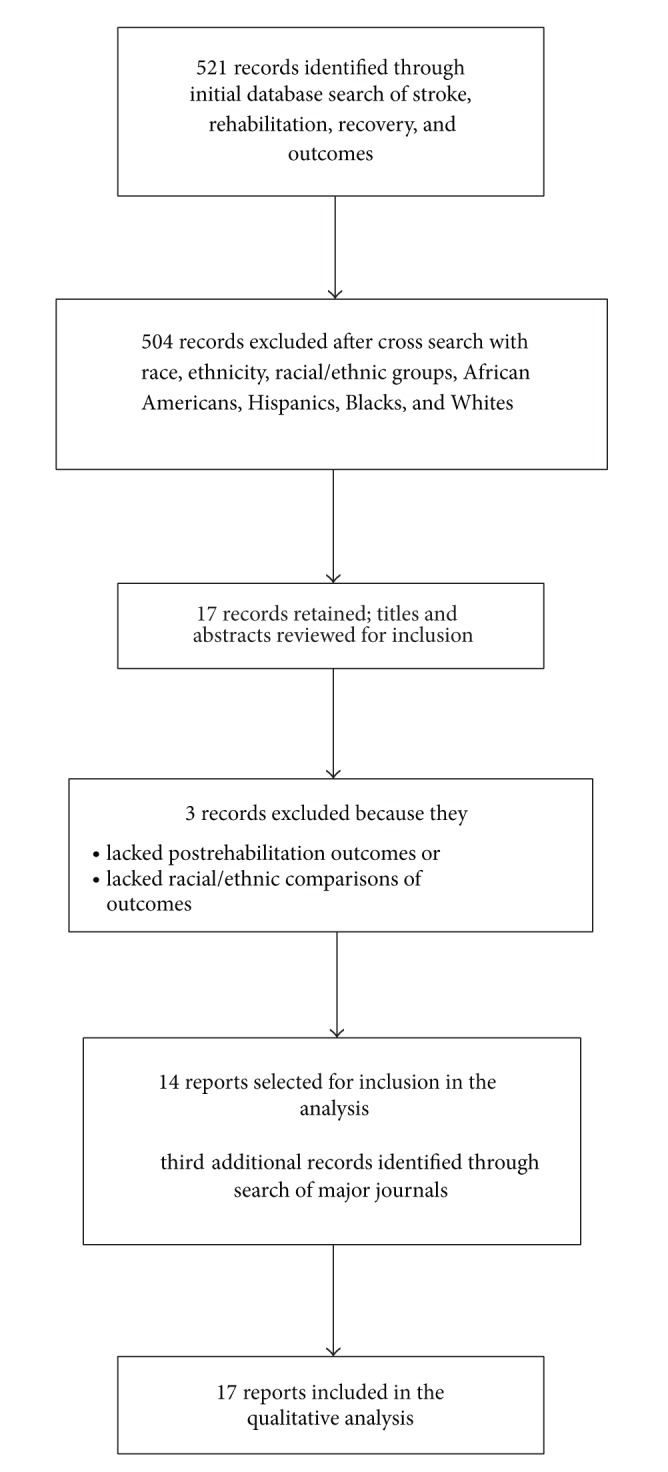
Flow chart of selected studies.

Five hundred and twenty-one unique reports were identified through an initial search with 504 being excluded after a cross search of terms related to race or ethnicity. Three authors (Charles Ellis, Hyacinth I. Hyacinth, and Jamie Beckett) independently assessed the full text or scientific abstract (for conference presentations) of the remaining 17 reports to identify eligible publications for inclusion in the final systematic review. Differences regarding study eligibility and need to proceed with data extraction were resolved by consensus. Three reports were excluded primarily due to lack of racial/ethnic comparisons of the outcomes. We then identified three additional reports following our search of the major journals dedicated to publishing research articles on stroke, rehabilitation, and racial or ethnic patterns of disease. In total, 17 studies including 429,108 stroke survivors met inclusion criteria and were included in the systematic review [11, 16–31]. See Figure 1 for flow chart of selection of studies.

### 3.1. Characteristics of Included Studies

Ninety-four percent (16/17) of studies explored outcomes disparities between Blacks and Whites and 53% (9/17) included comparisons between Blacks, White, and Hispanics. Ten of the 17 studies reported statistically significant racial/ethnic differences in poststroke rehabilitation outcomes [11, 17–20, 22, 23, 27, 28, 31]. The most widely reported outcome measure was the functional independence measure (FIM) [32]. Other rehabilitation outcomes reported included Rankin or Modified Rankin Scale [33], Stroke Impact Scale [34], Barthel Index [35], Mini Mental Status Exam [36], and the 6-minute walk test [37]. See Table 1 for a summary of the studies included in this review.

### 3.2. Functional Independence Measure (FIM)

The FIM is tool used to measure patient changes in functional ability during rehabilitation [32]. The FIM consists of 18 items that are assessed on seven-point ordinal scale, where higher scores indicate more independence in performing tasks. Thirteen of the 17 studies reported outcomes using the FIM including (a) total FIM (13 motor and 5 cognitive measures), (b) motor FIM (13 motor measures only), (c) cognitive FIM (5 cognitive measures only), (d) FIM gain (change scores between two measurement points), and (e) FIM efficiency (change scores between two measurement points divided by time needed to achieve change). Four studies reported outcomes at discharge and at least one follow-up point after discharge. Bhandari et al. examined functional improvement in 1002 stroke patients discharged from a community based inpatient rehabilitation facility between 1995 and 2001 [17]. Using multivariate models they found that Blacks achieved 7% less functional improvement at discharge compared to Whites (*P* = 0.02); however the difference did not remain at three months.

Berges and colleagues examined functional recovery in 990 Whites, Blacks, and Hispanics and found marginally significant differences at admission (*P* = 0.09), no differences at discharge (*P* = 0.15), and significant differences at 3-month follow-up (*P* = 0.01) with Whites (102.3) having higher FIM scores than Blacks (101.9) and Hispanics (92.0) [31]. These differences did not persist at 12-month follow-up (*P* = 0.18). Hinojosa et al. found higher motor FIM scores among Blacks when compared to Whites (*P* < 0.01) [23]. Over time the unadjusted motor FIM scores for Blacks and Puerto Ricans followed a curvilinear recovery trajectory and their 24-month scores were lower than their baseline scores. Finally, Putman and colleagues in a study of 732 patients in six US rehabilitation facilities found that, despite having lower total (*P* < 0.01) and cognitive FIM scores (*P* < 0.001) on admission, Whites compared to Blacks were also more likely to have a greater improvement in motor (*P* < 0.05) and cognitive (*P* < 0.01) FIM scores [27].

Three studies reported disparities in FIM scores. Moorthy and colleagues examined functional outcomes in 129 patients admitted to an acute rehabilitation unit from 2000 to 2001 [16]. They found an average FIM gain of 10.1 in Hispanics, 8.9 in Whites, and 7.1 in Blacks. They did not report statistical data related to differences by race/ethnicity and concluded that regardless of race/ethnicity or stroke subtype, lower admission FIM were associated with greater FIM gain. Chiou-Tan et al. found that race/ethnicity was associated with FIM gain (*P* = 0.014) and FIM efficiency scores (*P* = 0.035) [20]. In post hoc comparisons, Blacks (21.53) had the lowest FIM gain scores when compared to Hispanics (26.78) and Whites (21.70), even though there were no significant differences in the length of their rehabilitation stays (*P* = 0.26). Lower FIM gain scores among Blacks compared to Hispanics and Whites were also reported by Keng and colleagues in a study of 171 patients admitted to a stroke rehabilitation unit (*P* = 0.045) [19].

Four studies reported disparities in FIM efficiency scores. Ottenbacher and colleagues completed a retrospective analysis of 161,692 patients using the Uniform Data System for Medical Rehabilitation (USDMR). They found lower FIM efficiency scores among Blacks (1.53) compared to Whites (1.61) (*P* = 0.01) [22]. It was also noted that Blacks had lower admission and discharge FIM scores than Whites. Liu and colleagues found that, Black patients had lower functional status at discharge compared to Whites, Hispanics, North American natives, and Asians after adjusting to covariates [26]. They also found that Blacks were more likely to be discharged to community based settings despite lower function. In a 2-year prospective study of 670 admitted to an acute stroke rehabilitation facility within 30 days of their stroke, Rabadi et al. found that Whites (1.6) had lower change in cognitive FIM scores than Blacks (2.1), Hispanics (3.1), and Asians (3.5) (*P* = 0.028) [30]. There were no racial/ethnic differences in total FIM, ADL FIM and motor FIM scores at discharge. Finally, Asian race was associated with lower cognitive FIM scores in a study of 1908 moderately and severely impaired stroke patients by Wang and colleagues [29].

Three studies did not find significant racial disparities in FIM outcomes. Horn et al. did not consistently find Black White differences in total discharge FIM scores in 732 patients receiving inpatient stroke rehabilitation [24]. Disparities were not found regardless of whether they experienced a moderate (*P* = 0.767) or severe stroke (*P* = 0.518). However, Blacks with severe strokes did have lower change in total FIM scores (*P* = 0.019) and cognitive FIM scores (*P* = 0.006) than Whites. In a second study using the same cohort as the Horn study, Deutscher and colleagues measured disparities in motor function (accounting for patient characteristics, nontherapy ancillaries, therapy activities, and therapy interventions) and also did not report differences in discharge motor FIM scores between Whites and Blacks [25]. Finally, Wang and colleagues did not find disparities in outcomes between Blacks and Whites at discharge from an inpatient rehabilitation facility [29].

### 3.3. Modified Rankin Scale (MRS) and Barthel Index (BI)

The Modified Rankin Scale is a widely used clinical scale designed to measure the degree of disability or dependence in the completion of activities of daily living following stroke [33, 38]. The scale range is 0–6 with zero of no symptoms and six indicating death. The Barthel Index is an ordinal scale designed to measure performance in activities of daily living (ADL) ranging from dependent to independent. Horner and colleagues completed a secondary analysis of 598 patients (30% Black) who were followed for one year between 1995 and 1997 [11]. The Rankin score was completed at discharge and the Barthel Index was completed at follow-up. The two scales were standardized to allow assessment of relative change. Mixed models analyses indicated that Black patients recovered at a slower rate than Whites (*P* < 0.05) after controlling for sociodemographic characteristics, stroke type and severity, and cognitive function. The models further showed that, when rehabilitation was initiated after three days, only 16% of Blacks compared to 35% of Whites experienced substantial improvement (defined as a change of 25% points on the FIM) (*P* = 0.007). The authors concluded that delays in initiating rehabilitation were not directly responsible for worse functional recovery among Blacks. Linear regression models by Roth and colleagues also showed that Black race was associated with lower Barthel Index and Modified Rankin scores in a study of 112 stroke survivors 1 year after their event [28].

### 3.4. Stroke Impact Scale

The Stroke Impact Scale (SIS) is a self-report outcome measure designed to measure health related quality of life (HRQOL) [34]. Scores range from 0 to 100 for each domain with higher scores indicating higher HRQOL. Nichols-Larsen and colleagues measured HRQOL in 216 stroke survivors three to nine months after stroke and found that non-Whites who represented 29% of the sample reported poorer HRQOL in the physical domain (*P* = 0.0031) [18]. A second study by Roth and colleagues showed that Black race was associated with lower SIS memory, ADL, mobility, hand, and social domain scores approximately one year after stroke [28].

### 3.5. Other Measures

Hinson et al. examined cardiovascular fitness and ambulatory function in 118 hemiparetic stroke survivors being seen at an outpatient medical center [21]. They did not find statistically significant differences between Black men and women and White men and women on measures of cardiovascular fitness (VO_2  peak_) and the 6-minute walk test. Linear regression models by Roth and colleagues also showed that Black race was associated with lower Mini Mental Status Exam scores (*P* < 0.01) [28].

## 4. Discussion

The majority of the studies between at least two groups demonstrated that racial/ethnic minorities were less likely to achieve equivalent outcomes to their nonminority counterparts, despite both groups receiving rehabilitation. Black stroke survivors frequently achieved lower postrehabilitation discharge scores, lower gain/change scores, and lower efficiency scores. Although many of the reported studies included data suggesting racial/ethnic differences in outcomes, significant variability across studies in initial stroke severity at entry point of study, outcome measures, measurement time points, rehabilitation settings, and study design/type limits definitive conclusions regarding the presence and extent of such disparities. Even studies using the same outcomes completed measurements at different time points making a reconciliation of potential racial/ethnic differences more difficult.

In Table 2 we report the results of 11 studies that used the FIM to measure outcomes. The reported studies used both prospective and retrospective designs and univariate and multivariate analysis approaches. Racial/ethnic differences in outcomes were measured at admission, discharge, and follow-up time points along with FIM gain/change and efficiency scores. However, because of the variability in approaches, we experienced some difficulty in drawing definitive conclusions.

According to the National Institute of Neurological Disorders and Stroke, as many as two-thirds of all stroke survivors require rehabilitation to reduce functional limitations [39]. However little is known about the influence of race or ethnicity on outcomes after the receipt of poststroke rehabilitation services. Understanding racial/ethnic differences in poststroke rehabilitation outcomes remains difficult because of the complex number of factors related to the patient, the patient's rehabilitation setting, and the patient's home environment after rehabilitation. For example, controlling for stroke severity in specific racial/ethnic groups appears inconsistent across studies. This is important because race and stroke severity have been associated with differences in the use of rehabilitation, rehabilitation length of stays, and postrehabilitation discharge destinations [40–42]. These factors should be considered carefully in future studies designed to measure racial/ethnic differences in outcomes.

In our attempts to complete this review, we experienced a number of challenges. First, the study populations (age and stroke types) and rehabilitation settings were heterogeneous. In addition, in some studies we were unable to ascertain the length of rehabilitation stays, number and intensity of rehabilitation visits, and other factors known to drive rehabilitation utilization. Second, although a majority of studies reported FIM outcomes, there was inconsistency in the measurement time points and the specific FIM scores calculated (total, motor, cognitive, gain, change, and efficiency). Third, baseline data such as sociodemographic, clinical, and whether the patient experienced a recurrent stroke was absent in some studies particularly those completing secondary data analyses. Data could be misleading without factoring those important parameters into analysis. Fourth, rehabilitation system level issues such as practice patterns were not readily available.

Considering the aforementioned issues collectively, we believe further study of this issue is required particularly given the substantial burden of stroke. Such effort should include a focused study of stroke recovery from the stroke onset to specified time points after stroke, imaging and functional measure of stroke severity, global outcome measures, and domain specific performance measures. Other measures may include evaluating the effect of a stroke event on cerebrovascular CO_2_ reactivity and relationship to poststroke rehabilitation. With the increased use of tPA, studies should evaluate the impact of its use on postrehabilitation outcomes. Studies suggest that response to tPA may differ by race/ethnicity and it is possible that this difference may translate into a differential effect on prerehabilitation baseline outcomes and subsequently postrehabilitation outcomes [43–45]. In addition, access to care must be accounted for in studies of disparities in outcomes. Limited access to care among any racial/ethnic group can negatively impact the observed outcomes reported.

Finally the role of environmental exposures as it relates to poststroke response to rehabilitation might shed some light on the reason for racial disparity in poststroke rehabilitation recovery [46]. We also believe that greater attention should be given to knowledge and beliefs and attitudes that may influence rehabilitation participation and compliance. Negative attitudes and beliefs about stroke have been attributed to negative health behaviors which translate into poor short-term survival after stroke, increased risk of stroke recurrence, and poor stroke risk factor control [47, 48]. Similarly, individual emotional response to stroke and the presence of social networks after stroke must be carefully considered in studies of disparities in stroke-related outcomes.

In conclusion, despite the heterogeneous nature of most of the available studies in design, measures, and subject selection, there is an argument to be made for the existence of a racial disparity in poststroke rehabilitation outcomes. Additionally, the nature of the available evidence plus the need for more targeted/individualized intervention necessitate the conduct of well-designed, adequately powered, large-scale prospective studies to examine racial/ethnic differences in poststroke outcomes and definitively answer questions about racial/ethnic differences in poststroke rehabilitation outcomes.

## Figures and Tables

**Table 1:** Summary of studies reporting racial or ethnic differences in rehabilitation outcomes.

Study [ref. no.]	Data source	Sample	Outcome	Time of measure	Severity controlled	Results	*P* value	Comment
Horner et al., 2003 [11]	Patients hospitalized in 9 VAMCs 1995–1997	738 (31.2% Black)	Rankin % change	Admit-D/C	Yes	Improvement after 3 days of rehab 16% of Blacks 35% of Whites	0.007	Low income Blacks had worse recovery; delay in rehab initiation had greater impact on Blacks

Moorthy et al., 2004 [16]	Patients in IP rehab	^‡^Black: 56 White: 55 Hispanic: 18	FIM	Admit-D/C	No	Improvement at D/C ^‡^Blacks: 7.1 White: 8.9 Hispanic: 10.1	NR	Small statistically significant differences in FIM gain scores among ethnic groups

Bhandari et al., 2005 [17]	Patients in community based IP rehab facility 1995–2001	Black: 419 White: 421 Hispanic: 33 Asian: 96 Other: 33	FIM	D/C-3 months	No	Improvement at D/C Blacks: 1.9 points (7% less) Improvement at 3 months Asians 16% less than Whites	0.02 0.005	Blacks with less improvement but more likely to be discharged home

Nichols-Larsen et al., 2005 [18]	EXCITE trial	White: 153 Non-White: 63	SIS	3–9 months after stroke	No	Non-Whites reported lower HRQOL in the physical domain	0.003	

Keng et al., 2005 [19]	Patients in urban community hospital	^‡^Black: 83 ^∞^White: 20 Hispanic: 68	FIM	Admit-D/C	No	Improvement at D/C Hispanic: 31.4 Caucasian: 28.4 Blacks: 18.9		FIM gain higher for Hispanic and Caucasian but only significant between Hispanics and Blacks (*P* = 0.045)

Chiou-Tan et al., 2006 [20]	UDSMR data 2000–2003	Black: 83 White: 20 Hispanic: 68	FIM	Admit-D/C	No	Improvement at D/C Black: 21.53 White: 21.70 Hispanic: 26.78 Efficiency to D/C Black: 1.43 White: 1.20 Hispanic: 1.70	0.014 0.035	Blacks with higher scores on admission (68.89), compared to Whites (66.50) and Hispanics (58.89) (*P* = 0.005)

Hinson et al., 2007 [21]	Patients seen at outpatient medical center	Black: 66 White: 52	VO_2 peak_, 6-min walk test, and 30 ft walking velocity	Admit only	No	Walking velocity BM-.60 BF-.50 WM-.61 WF-.41 6 min walk test BM: 223 BF: 198 WM: 226 WF: 157 VO_2 peak_ BM: 15 BF: 11.5 WM: 15.1 WF: 12.3	NS NS NS	Reported as BM: Black male; BF: Black female; WM: White male; WF: White female

Ottenbacher et al., 2008 [22]	UDSMR data; IP rehab in 2002-2003	Black: 25,334 White: 123,537 Hispanic: 7,994 Other: 4,827	FIM	Admit-D/C	No	D/C FIM Black: 80.23^†^ White: 81.54 Hispanic: 79.43^†^ Other: 81.77 Efficiency to D/C Black: 1.53^†^ White: 1.61 Hispanic: 1.57 Other: 1.59	^†^0.01 ^†^0.01	Differences in functional status across race groups were related to age. White was reference group.

Hinojosa et al., 2009 [23]	Veterans in the US and Puerto Rico	^‡^Black: 30 ^∞^White: 42 Puerto Rican: 49	FIM	D/C 1 months 6 months 12 months 18 months 24 months	No	D/C motor FIM Black: 80.94 Caucasian: 78.29 Puerto Rican: 67.66	0.000	In HLM models and controlling for time, Blacks had average FIM 4.66 points higher than Whites (*P* < 0.01) and Puerto Ricans had FIM 5.64 points lower than Whites (*P* < 0.05)

Horn et al., 2010 [24]	Six US inpatient rehabilitation facilities	Black: 239 White: 493	FIM	Admit-D/C	No	Improvement at D/C Moderate stroke Blacks: 24.9 Whites: 26.9 Severe stroke Blacks: 28.1 Whites: 32.2	0.066 0.019	Moderate and severe strokes analyzed separately No Black/White differences found in unadjusted stroke rehabilitation outcomes

Deutscher et al., 2010 [25]	Six US inpatient rehabilitation facilities	Black: 239 White: 493	FIM	Admit-D/C	No	Blacks with lower discharge FIM (In OLS models using patient variables, nontherapy ancillaries, and use of PT/OT)	0.015	

Liu et al., 2010 [26]	Medicare assessment and claims data	Black: 33,639 White: 216,664 Asian: 3,157 Hispanic: 4,575 North American Natives: 839	FIM: 12	Admit-D/C	No	Blacks had lower functional status than Whites after adjusting for covariates	NR	

Putnam et al., 2010 [27]	Six US inpatient rehabilitation facilities	^‡^Blacks: 239 Whites: 493	FIM	Admit-D/C	No	Improvement at D/C Motor FIM Whites: 24.5 Blacks: 22.6 Cognitive FIM Whites: 4.9 Blacks: 3.7	<0.05 <0.01	Significant differences among those with severe stroke; no differences among moderate stroke

Roth et al., 2011 [28]	Patients enrolled in REGARDS study	Blacks: 40 Whites: 72	BI MRS SIS MMSE	Admit-1 year	No	Race-Adjusted Coefficients BI MMSE SIS memory SIS ADL SIS mobility SIS hand SIS social	<0.05 <0.01 <0.01 <0.01 <0.05 <0.05 <0.05	Blacks showed greater deficits on multiple 1-year outcome measures

Wang et al., 2011 [29]	Inpatient rehabilitation hospital 2002–2006	1908 Black: 13.5% White: 63.9% Hispanic: 8.4% Asian: 14.1%	FIM	Admit-D/C	No	Improvement at D/C Severe Impairment Cognitive FIM Black (−0.09) Hispanic (0.42) Asian (−0.78)	0.7893 0.2963 0.0180	Moderate and severe strokes analyzed separately

Rabadi et al., 2012 [30]	Acute stroke rehabilitation unit	Black: 115 White: 504 Hispanic: 38 Asian: 13	FIM	Admit-D/C	No	Improvement at D/C Cognitive FIM Whites: 1.6 Blacks: 2.1 Hispanics: 3.1 Asians: 3.5	0.028	No significant differences noted in total FIM, FIM-ADL, or motor FIM

Berges et al., 2012 [31]	11 US IP facilities	Black: 150 White: 783 Hispanic: 57	FIM	Admit-D/C 3 months 12 months	No	Improvement at D/C Blacks: 82.9 Whites: 78.8 Hispanics: 80.3 Improvement at 3-month follow-up Blacks: 101.9 Whites: 102.3 Hispanics: 92.0 Improvement at 12-month follow-up Blacks: 105.0 Whites: 105.9 Hispanics: 98.7	0.15 0.01 0.18	No significant racial differences on admission

Admit: admission; D/C: discharge; NR: not reported; NS: not significant; IP: inpatient; IRH: inpatient rehab hospital; FIM: functional independence measure; BI: Barthel Index; MRS: Modified Rankin Scale; SIS: Stroke Impact Scale; MMSE: Mini Mental Status Exam; IRF-PAI: inpatient rehabilitation facilities-patient assessment instrument; HRQOL: health related quality of life; UDSMR: Uniform Data System for Medical Rehabilitation; EXCITE: extremity constraint induced therapy evaluation; REGARDS: reasons for geographic and racial differences in stroke.

^‡^Reported as African American in study.

^∞^Reported as Caucasian in study.

^†^Refers to group statistically significant.

**Table 2:** Select studies reporting FIM admission, discharge, gain, and efficiency scores.

Study [ref. no.]	FIM admission	FIM discharge	FIM gain	FIM efficiency	Follow-up
Moorthy et al., 2004 [16]			Blacks: 7.1 White: 8.9 Hispanic: 10.1		

Bhandari et al., 2005 [17]	Blacks: 57.24 Whites: 58.26 (ref) Hispanics: 57.73 Asian Americans: 53.56 Others: 51.48 (*P* < 0.05)		FIM gain is 1.9 points lower for Blacks than Whites (*P* = 0.02)		Three months Blacks 1.5 points > Whites (*P* = 0.30); Asians 6.3 points >Whites (*P* ≤ 0.01)

Keng et al., 2005 [19]			Black: 18.9 White: 28.4 Hispanic: 31.4 (*P* = 0.045)		

Chiou-Tan et al., 2006 [20]	Blacks: 68.89 Whites: 66.50 Hispanics: 58.89 (*P* = 0.005)	Blacks: 90.42 Whites: 88.20 Hispanics: 85.37 (*P* = 0.314)	Blacks: 21.53 Whites: 21.70 Hispanics: 26.78 (*P* = 0.014)	Blacks: 1.43 Whites: 1.20 Hispanics: 1.70 (*P* = 0.035)	

Ottenbacher et al., 2008 [22]	Blacks: 58.01 Whites: 58.82 (ref) Hispanics: 55.82 Others: 57.06 (*P* < 0.01)	Blacks: 80.23 Whites: 81.54 (ref) Hispanics: 79.43 Others: 81.77 (*P* < 0.01)		Blacks: 1.53 Whites: 1.61 (ref) Hispanics: 1.57 Other: 1.59 (*P* < 0.01)	

Hinojosa et al., 2009 [23]		Motor FIM: Blacks: 80.8 Whites: 76.1 Puerto Rican: 70.5			12 months Blacks: 86.2 Whites: 81.1 Puerto Rican: 76.7

Horn et al. 2010 [24]	Moderate: Blacks: 73.5 Whites: 71.9 (*P* = 0.118) Severe: Blacks: 49.0 Whites: 43.4 (*P* < 0.001)	Moderate: Blacks: 98.4 Whites: 98.8 (*P* = 0.767) Severe: Blacks: 77.3 Whites: 75.7 (*P* < 0.518)	Moderate: Blacks: 21.8 Whites: 23.0 (*P* = 0.173) Severe: Blacks: 28.1 Whites: 32.2 (*P* < 0.019)		

Liu et al., 2010 [26]		Blacks: 46.9 North American natives: 51.0			

Putman et al., 2010 [27]	Blacks: 63 Whites: 58 (*P* < 0.01)	Blacks: 89.1 Whites: 88.0	Lower motor FIM increase (*P* < 0.05) and cognitive FIM increase (*P* < 0.001) among Blacks		

Rabadi et al., 2012 [30]	Blacks: 62.5 Whites: 59.6 Hispanics: 58.6 Asians: 60.8 (*P* = 0.50)		Blacks: 19.2 Whites: 17.8 Hispanics: 20.4 Asians: 20.8 (*P* = 0.039)		

Berges et al., 2012 [31]	Blacks: 58.1 Whites: 54.4 Hispanics: 55.4 (*P* = 0.09) Unadjusted	Blacks: 82.9 Whites: 78.8 Hispanics: 80.3 (*P* = 0.15) Unadjusted			Three months Blacks: 101.9 Whites: 102.3 Hispanics: 92.0 (*P* = 0.01) 12 months Blacks: 105.0 Whites: 105.9 Hispanics: 98.7 (*P* = 0.18)

## References

[1] Go AS, Mozaffarian D, Roger VL (2013). Heart disease and stroke statistics-2013 update: a Report from the American Heart Association. *Circulation*.

[2] National Stroke Association, African Americans and Stroke. http://www.stroke.org/site/PageServer?pagename=AAMER.

[3] White H, Boden-Albala B, Wang C (2005). Ischemic stroke subtype incidence among whites, blacks, and Hispanics: the northern Manhattan study. *Circulation*.

[4] Qian F, Fonarow GC, Smith EE (2013). Racial and ethnic differences in outcomes in older patients with acute ischemic stroke. *Circulation: Cardiovascular Quality and Outcomes*.

[5] Kleindorfer DO, Khoury J, Moomaw CJ (2010). Stroke incidence is decreasing in whites but not in blacks: a population-based estimate of temporal trends in stroke incidence from the greater cincinnati/northern kentucky stroke study. *Stroke*.

[6] Horner RD, Matchar DB, Divine GW, Feussner JR (1991). Racial variations in ischemic stroke-related physical and functional impairments. *Stroke*.

[7] Jones MR, Horner RD, Edwards LJ (2000). Racial variation in initial stroke severity. *Stroke*.

[8] Kuhlemeier KV, Stiens SA (1994). Racial disparities in severity of cerebrovascular events. *Stroke*.

[9] Gaines K, Burke G (1995). Ethnic differences in stroke: black-White differences in the United States population. *Neuroepidemiology*.

[10] Gillum RF (1999). Stroke mortality in blacks: disturbing trends. *Stroke*.

[11] Horner RD, Swanson JW, Bosworth HB, Matchar DB (2003). Effects of race and poverty on the process and outcome of inpatient rehabilitation services among stroke patients. *Stroke*.

[12] Kleindorfer D (2009). Sociodemographic groups at risk: race/ethnicity. *Stroke*.

[13] Centers for Disease Control and Prevention (CDC) (2005). Differences in disability among black and white stroke survivors—United States, 2000-2001. *Morbidity and Mortality Weekly Report*.

[14] Stansbury JP, Jia H, Williams LS, Vogel WB, Duncan PW (2005). Ethnic disparities in stroke: epidemiology, acute care, and postacute outcomes. *Stroke*.

[15] Cruz-Flores S, Rabinstein A, Biller J (2011). Racial-ethnic disparities in stroke care: the American experience: a statement for healthcare professionals from the American Heart Association/American Stroke Association. *Stroke*.

[16] Moorthy P, Xiaoqi L, Noser E, Tran T (2004). Poster 276 stroke rehabilitation outcomes between ethnic groups. *Archives of Physical Medicine and Rehabilitation*.

[17] Bhandari VK, Kushel M, Price L, Schillinger D (2005). Racial disparities in outcomes of inpatient stroke rehabilitation. *Archives of Physical Medicine and Rehabilitation*.

[18] Nichols-Larsen DS, Clark PC, Zeringue A, Greenspan A, Blanton S (2005). Factors influencing stroke survivors’ quality of life during subacute recovery. *Stroke*.

[19] Keng MJ, Graves DE, Chan KT, Chiou-Tan FY (2005). Ethnic differences in FIM gain for indigent patients undergoing stroke inpatient rehabilitation. *The American Journal of Physical Medicine and Rehabilitation*.

[20] Chiou-Tan FY, Keng MJ, Graves DE, Chan K-T, Rintala DH (2006). Racial/ethnic differences in FIM scores and length of stay for underinsured patients undergoing stroke impatient rehabilitation. *The American Journal of Physical Medicine and Rehabilitation*.

[21] Hinson HE, Patterson SL, Macko RF, Goldberg AP (2007). Reduced cardiovascular fitness and ambulatory function in black and white stroke survivors. *Ethnicity and Disease*.

[22] Ottenbacher KJ, Campbell J, Kuo Y-F, Deutsch A, Ostir GV, Granger CV (2008). Racial and ethnic differences in postacute rehabilitation outcomes after stroke in the united states. *Stroke*.

[23] Hinojosa MS, Rittman M, Hinojosa R, Rodriguez W (2009). Racial/ethnic variation in recovery of motor function in stroke survivors: role of informal caregivers. *Journal of Rehabilitation Research and Development*.

[24] Horn SD, Deutscher D, Smout RJ, Dejong G, Putman K (2010). Black-white differences in patient characteristics, treatments, and outcomes in inpatient stroke rehabilitation. *Archives of Physical Medicine and Rehabilitation*.

[25] Deutscher D, Horn SD, Smout RJ, Dejong G, Putman K (2010). Black-white disparities in motor function outcomes taking into account patient characteristics, nontherapy ancillaries, therapy activities, and therapy interventions. *Archives of Physical Medicine and Rehabilitation*.

[26] Liu EY, Beutsch A, Drake H (2010). Poster 88: racial and ethnic differences in outcomes of inpatient stroke rehabilitation in the United States. *Archives of Physical Medicine and Rehabilitation*.

[27] Putman K, Horn S, Smout R (2010). Racial disparities in stroke functional outcomes upon discharge from inpatient rehabilitation facilities. *Disability and Rehabilitation*.

[28] Roth DL, Haley WE, Clay OJ (2011). Race and gender differences in 1-year outcomes for community-dwelling stroke survivors with family caregivers. *Stroke*.

[29] Wang H, Camicia M, Terdiman J, Hung Y-Y, Sandel ME (2011). Time to inpatient rehabilitation hospital admission and functional outcomes of stroke patients. *PM and R*.

[30] Rabadi MH, Rabadi FM, Hallford G, Aston CE (2012). Does race influence functional outcomes in patients with acute stroke undergoing inpatient rehabilitation?. *The American Journal of Physical Medicine and Rehabilitation*.

[31] Berges I-M, Kuo Y-F, Ottenbacher KJ, Seale GS, Ostir GV (2012). Recovery of functional status after stroke in a tri-ethnic population. *PM and R*.

[32] Keith RA, Granger CV, Hamilton BB, Sherwin FS (1987). The functional independence measure: a new tool for rehabilitation. *Advances in Clinical Rehabilitation*.

[33] Rankin J (1957). Cerebral vascular accidents in patients over the age of 60. II. Prognosis. *Scottish Medical Journal*.

[34] Duncan PW, Wallace D, Lai SM, Johnson D, Embretson S, Laster LJ (1999). The stroke impact scale version 2.0: evaluation of reliability, validity, and sensitivity to change. *Stroke*.

[35] Mahoney FI, Barthel DW (1965). Functional evaluation: the barthel index. *Maryland State Medical Journal*.

[36] Folstein MF, Folstein SE, McHugh PR (1975). ’Mini mental state’. A practical method for grading the cognitive state of patients for the clinician. *Journal of Psychiatric Research*.

[37] Balke B (1963). *A Simple Field Test for the Assessment of Physical Fitness*.

[38] Van Swieten JC, Koudstaal PJ, Visser MC, Schouten HJA, Van Gijn J (1988). Interobserver agreement for the assessment of handicap in stroke patients. *Stroke*.

[39] National Institute of Neurological Disorders and Stroke-Department of Health and Human Services, Post-Stroke Rehabilitation. NIH Publication No. 11-1846. http://stroke.nih.gov/documents/Post-Stroke_Rehabilitation_english_brochure.pdf.

[40] Elwood D, Rashbaum I, Bonder J (2009). Length of stay in rehabilitation is associated with admission neurologic deficit and discharge destination. *PM and R*.

[41] Onukwugha E, Mullins CD (2007). Racial differences in hospital discharge disposition among stroke patients in Maryland. *Medical Decision Making*.

[42] Ellis C, Breland HL, Egede LE (2008). Reviews and commentaries racial/ethnic differences in utilization of post-stroke rehabilitation services: a systematic review. *Ethnicity and Disease*.

[43] Mishra NK, Mandava P, Chen C Influence of racial differences on outcomes after thrombolytic therapy in acute ischemic stroke.

[44] Ueshima S, Matsuo O (2002). The differences in thrombolytic effects of administrated recombinant t-PA between Japanese and Caucasians. *Thrombosis and Haemostasis*.

[45] Sane DC, Stump DC, Topol EJ (1991). Racial differences in responses to thrombolytic therapy with recombinant tissue-type plasminogen activator. Increased fibrin(ogen)olysis in blacks. *Circulation*.

[46] Kim J-M, Stewart R, Park M-S (2012). Associations of BDNF genotype and promoter methylation with acute and long-term stroke outcomes in an East Asian cohort. *PLoS ONE*.

[47] Lewis SC, Dennis MS, O’Rourke SJ, Sharpe M (2001). Negative attitudes among short-term stroke survivors predict worse long-term survival. *Stroke*.

[48] Morgenstern LB, Sánchez BN, Skolarus LE (2011). Fatalism, optimism, spirituality, depressive symptoms, and stroke outcome: a population-based analysis. *Stroke*.

